# Identification of skipjack tuna (*Katsuwonus pelamis*) pelagic hotspots applying a satellite remote sensing-driven analysis of ecological niche factors: A short-term run

**DOI:** 10.1371/journal.pone.0237742

**Published:** 2020-08-20

**Authors:** Robinson Mugo, Sei-Ichi Saitoh, Hiromichi Igarashi, Takahiro Toyoda, Shuhei Masuda, Toshiyuki Awaji, Yoichi Ishikawa

**Affiliations:** 1 Regional Center for Mapping of Resources for Development, Nairobi, Kenya; 2 Arctic Research Center, Hokkaido University, Hokkaido, Japan; 3 Information Engineering Program, Japan Agency for Marine-Earth Science and Technology (JAMSTEC), Yokosuka, Japan; 4 Oceanography and Geochemistry Research Department, Meteorological Research Institute, Japan Meteorological Agency, Yokosuka, Japan; 5 Research Institute for Global Change (RIGC), Japan Agency for Marine-Earth Science and Technology (JAMSTEC), Yokosuka, Japan; 6 Department of Geophysics, Kyoto University, Kyoto, Japan; Hawaii Pacific University, UNITED STATES

## Abstract

Skipjack tuna (SJT) pelagic hotspots in the western North Pacific (WNP) were modelled using fishery and satellite remotely sensed data with Ecological Niche Factor Analysis (ENFA) models. Our objectives were to model and predict habitat hotspots for SJT and assess the monthly changes in sub-surface temperatures and mixed layer depths at fishing locations. SJT presence-only monthly resolved data, sea surface temperature, chlorophyll-a, diffuse attenuation coefficient, sea surface heights and surface wind speed were used to construct ENFA models and generate habitat suitability indices using a short-term dataset from March-November 2004. The suitability indices were then predicted for July-October (2007 and 2008). Monthly aggregated polygons of areas fished by skipjack tuna pole and line vessels were also overlaid on the predicted habitat suitability maps. Distributions of sub-surface temperatures and mixed layer depths (MLD) at fishing locations were also examined. Our results showed good fit for ENFA models, as indicated by the absolute validation index, the contrast validation index and the continuous Boyce index. The predicted hotspots showed varying concurrences when compared with 25-degree polygons derived from fished areas. Northward shifts in SJT hotspots corresponded with declining MLDs from March to September. The MLDs were shallower in summer and deeper in autumn and winter months. The habitat hotspots modeled using ENFA were consistent with the known ecology and seasonal migration pattern of SJT. The findings of this work, derived from a short-term dataset, enable identification of SJT hotspots in the WNP, thus contributing valuable information for future research on SJT habitat prediction models.

## Introduction

Pelagic biological hotspots in the ocean are areas of elevated productivity, created by physical processes or features such as upwelling, fronts, eddies, bathymetry, or river discharge among other factors [1–6]. They are characterized by high concentrations of organisms, that attract large numbers of top predators, thus becoming fishery targets [7, 8]. Pelagic hotspots are now an important dimension in fishery forecasting, marine resource management and conservation, primarily in the design of dynamic marine conservation zones [9, 10]. Hotspots have been the subject of intense scientific research, often driven by the state-of-the-art tools such as remote sensing, remotely operated vehicles, tags, ocean circulation models, and habitat models [11]. Due to the heterogeneity and spatial extent of the world oceans, new tools and techniques will continue to be tested in efforts to understand pelagic biological hotspots. One of the pressing issues entails developing robust tools to identify biological hotspots and predict their spatial and temporal dynamics.

Skipjack tuna (*Katsuwonus pelamis*) is one of the widely fished tunas inhabiting the upper mixed layer [12, 13], an opportunistic predator feeding mainly on pelagic fishes, squids, a variety of crustaceans and young skipjacks [14, 15]. These tunas cover large distances in search of areas with high concentrations of forage [12]. In the western North Pacific, they migrate north from spring to summer, and south at the onset of winter, in a seasonal migration pattern associated with feeding [16, 17]. During migration, the fish track highly productive areas associated with physical oceanographic features involving sea surface temperature and ocean color gradients, eddies and warm streamers (filaments of warm water entrained into cooler waters) [8, 18–20]. These habitat associations are useful for modeling skipjack tuna aggregations, and for hypothesizing how ocean warming will affect the distribution of tuna around such oceanographic features.

The Ecological Niche Factor Analysis (ENFA) is a multivariate approach that computes suitability functions by comparing the species distribution in its environmental space, with the environmental conditions potentially available to the species [21]. Suitability functions express the relationship between the species occurrence and the environmental space that it occupies. ENFA is based on the computation of the factors explaining the major part of species environmental distribution. The ENFA approach is a presence-only data model, which does not use absence data, often associated with false absences and insufficient sampling effort [22]. For tuna, which are highly mobile, and whose majority of available distribution datasets are obtained from fisheries, eliminating biases associated with fishing strategies and the assumptions that null catches represent species absences can be challenging [23]. The ENFA is in a family of species distribution models [24] that can be used to explain habitat utilization of a species, by estimating its niche using occurrence records and environmental predictor layers [21]. We chose ENFA for four reasons: (i) it does not require absence data, and is able to immediately establish and interpret correlations among variables [21]; (ii) environmental grids are matched to species occurrence grids of the same resolution, hence facilitating a pixel to pixel match of species occurrence and environmental layers; (iii) results are easy to compare with other methods, which also output normalized suitability maps; and (iv) is able to predict future habitats based on models fitted from “current conditions” and similar environmental predictors from future scenarios [21]. The ENFA approach has been applied widely in terrestrial ecology [25, 26] and applications in marine studies using remotely sensed data are increasing [27–30].

Recent approaches in modeling marine top predators’ habitat are addressing the horizontal and vertical habitat utilization, by integrating oceanographic data from remotely-sensed sources and electronic tags [31–33]. These approaches provide a holistic understanding of an organism’s habitat from a 3-dimensional perspective. In the western North Pacific, several studies [3, 8, 9, 33, 34] have explained the ecological significance of sea surface temperature, chlorophyll-a, currents and ocean dynamic topography on tuna pelagic habitats using species distribution models and remotely sensed data. Our work presents preliminary runs to explore the relationship between tuna aggregation (hotspots) and oceanographic features (thermal or ocean color fronts, eddies, warm currents and their streamers) [17, 18] using multiple parameters as indicators of hotspot formation in a species distribution model. The objectives of our work were to: (i) model and predict habitat hotspots for skipjack tuna using ENFA and satellite remotely sensed data, and (ii) determine the sub-surface temperatures and mixed layer depths at fishing locations, and thus complement the explanatory information at the fishing locations quantified using satellites.

## Materials and methods

### Study area

**Fig 1:**
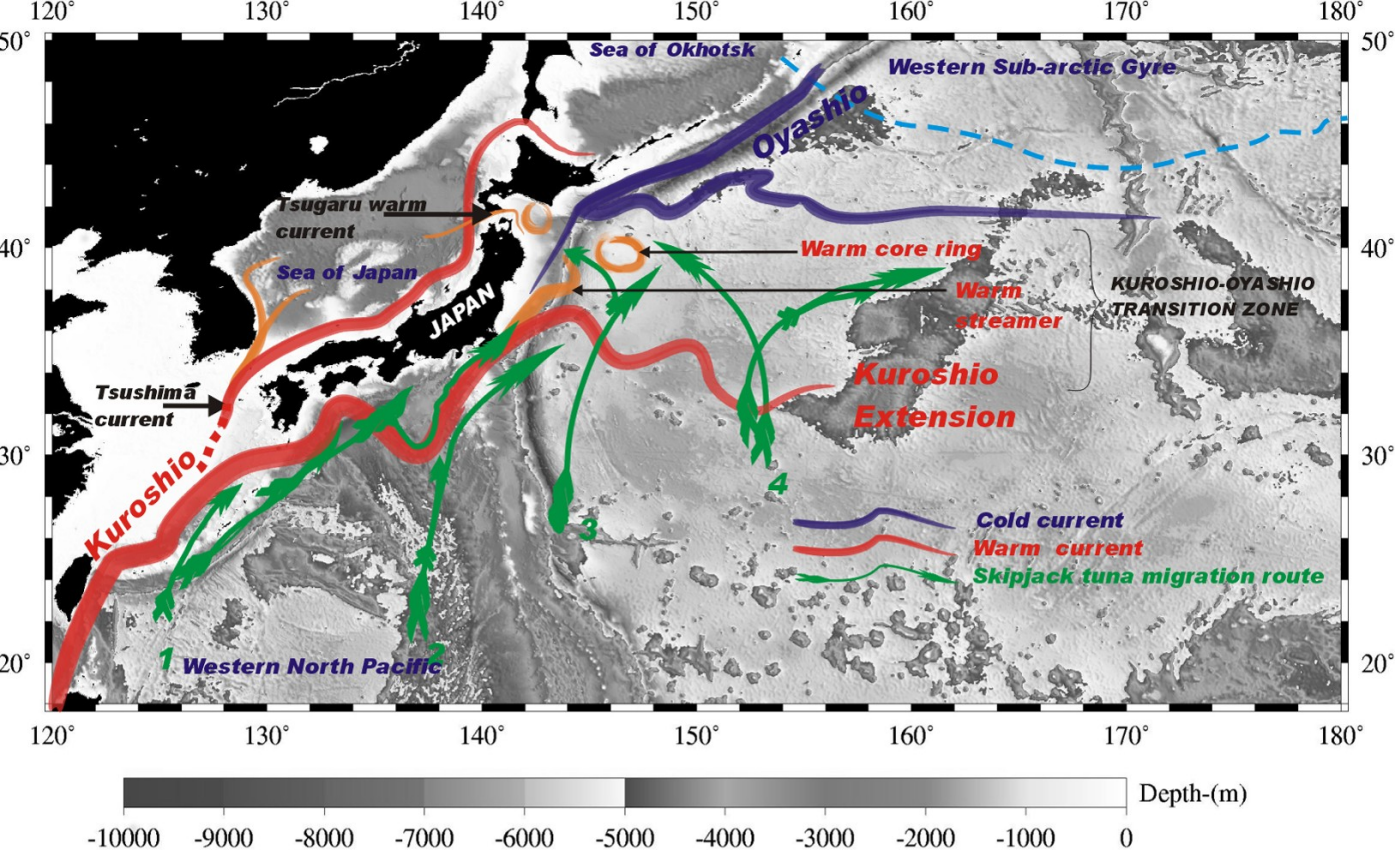
The study area, showing the western North Pacific (18-50^o^ N and 125-180^o^ E), the major currents and migration routes of skipjack tuna as described in [17].

The physical oceanography of the western North Pacific (18-50^o^ N and 125-180^o^ E) is shaped by three main currents: the Oyashio Current, the Kuroshio Current, and the Tsugaru Warm Current (Fig 1, Table 1). The Oyashio waters flow southward [35], transporting low temperature, low salinity and nutrient rich waters to the sub-tropical gyre [36], and forming two southward tongue-shaped intrusions off Honshu, known as the First and Second Oyashio Intrusions [37, 38]. A warm core ring (WCR) originating from the northward movement of the ring produced by the Kuroshio separates the meanders [39]. The southern limit of sub-polar waters is referred to as the Oyashio Front [40]. The Kuroshio Current assumes three major paths south of Japan, which affect formation of pelagic fisheries [37]. The behavior of the Kuroshio Extension, warm streamers and WCRs in the Transition Zone affect fishing ground formation [18, 41]. The vertical structure in the western North Pacific is characterized by shallow mixed layer depths in spring and summer and deeper mixed layer depths in late autumn and early winter [42, 43].

**Table 1:** Properties of major currents and water masses in the study area.

Water mass/zone	Properties	Citation
Kuroshio Current (KC)	Low density, nutrient poor, warm and high salinity	[38, 40]
Kuroshio Extension (KE)	Large-amplitude meanders, energetic eddies, high eddy kinetic energies	[46]
Oyashio Current (OC)	Temperatures lower than 5°C at 100m depth, high nutrients, fishing ground for several sub-arctic species and sub-tropical migrants	[18, 36]
Kuroshio-Oyashio Transition Zone (KOTZ)	Confluence waters of KC and OC	[35, 44]
Tsugaru Warm Current (TC)	Warm and saline water, from Tsushima Current	[40]

The distribution and density of phytoplankton and zooplankton populations in the western North Pacific is influenced by the ocean circulation and behavior of currents [37]. High densities of phytoplankton subsequently support large populations of zooplankton, which are fed upon by smaller nekton [35, 40, 44]. Skipjack tuna and other pelagic predators are attracted to such areas of high productivity to forage on the small organisms (squids, crustaceans, and fishes) [9, 12, 17, 41]. Fishers of tuna in the western North Pacific locate areas with dense aggregations of tuna, by tracking oceanic fronts, upwelling zones, and edges of large eddies [34, 45].

### Fishery data

Two different fishery datasets were used as occurrence records for modelling and polygons for qualitative validation. First, daily skipjack tuna catches from March to November 2004 were obtained from the Ibaraki Prefecture Fisheries Research Station. For the ENFA model, daily fishing data were digitized, compiled into monthly composites and converted into 0.25^o^ resolution grids [21]. Re-gridding was necessary to ensure that the fishery data matched the resolution of the environment grids, and resulted in a total of 15663 valid grid cells. Second, a 5x5 degree monthly aggregated skipjack tuna pole and line fishery catch data were downloaded from the Western and Central Pacific Fisheries Commission website (http://www.wcpfc.int/public-domain; last accessed in December 2018). The data (55 points in 2007 and 66 in 2008) were gridded into 5x5 degree grids, for all locations where the catch per unit effort was above zero, and the grid maps were converted into polygons of areas fished between July-October, in 2007 and 2008.

### Environmental data

A monthly environment database consisting of sea surface temperature (SST), sea surface chlorophyll-a (SSC), diffuse attenuation coefficient (Kd490), sea surface height (SSH), and wind speed (WS) was compiled from a variety of sources (Table 2). These five parameters were selected because of their relevance as descriptors of skipjack tuna habitat, and their capacity to reflect changes in climatic patterns [47, 48]. SST data are an important indicator of distribution patterns of tuna, which prefer foraging close to thermal fronts, and also migrating within physiologically tolerable temperatures, above 15 ^o^C [9, 12]. Sea surface chlorophyll concentration measured by satellites provides information on ocean productivity [19], which can reveal surface frontal and eddy-like features that are not always evident in SST maps [49, 50]. Due to the elevated productivity around these features, they attract large schools of tuna, which aggregate around them to feed on lower trophic level organisms [3, 41]. The diffuse attenuation coefficient is a good indicator of turbidity, the depth of the euphotic zone, and ultimately the maximum depth of primary production of the ocean [49, 51]. Tuna forage by sight and extremely turbid waters are unsuitable for foraging [52, 53] while extremely oligotrophic waters will contain little forage. The SSH data are an indicator of ocean dynamic topography, which provides information on movement of water masses, and by extension the flow of heat and nutrients, which subsequently influence productivity [43]. The ocean surface is also influenced by surface winds, which drive physical processes such as mixing of the upper layer and upwelling [54].

**Table 2:** Data layers used, their resolutions and sources.

Data	Resolution	Source	Agency
SST-v2, AVHRR-AMSRE	0.25	ftp://eclipse.ncdc.noaa.gov/pub/OI-daily-v2/IEEE	NOAA
SSC	0.05	https://oceancolor.gsfc.nasa.gov/l3/	NASA
SSH-dt, updated and merged, global	0.25	ftp.aviso.oceanobs.com/pub/oceano/AVISO/SSH/duacs/Data_Test/README.txt	AVISO
KD490	0.05	https://oceancolor.gsfc.nasa.gov/l3/	NASA
Wind speed-v6, SSM/I, DMSP F13	0.25	http://images.remss.com/ssmi/ssmi_data_monthly.html?&keep=0	REMSS
MLD	0.25	4D-VAR	JAMSTEC
SST*u*	0.25	4D-VAR	JAMSTEC

Daily resolved optimally interpolated sea surface temperature global data were downloaded from the National Oceanic Atmospheric Administration’s National Climatic Data Center (Table 2), for the period 2004 to 2008. These data provided better coverage of the fishing locations because the effect of missing data due to clouds is eliminated. Monthly Aqua-MODIS ~4km standard mapped images (2004 to 2008) of SSC and Kd490 data were downloaded from the ocean color data portal (Table 2).

The weekly SSH data were downloaded from the Archiving, Validation and Interpretation of Satellite Oceanographic data portal (Table 2) and processed using the public reading routines, from which monthly averages were made. In addition, monthly averaged geostrophic velocity vectors were used to indicate the magnitude and direction of flow along the Kuroshio Extension. Monthly averaged wind speed global images were downloaded from Remote Sensing Systems website (Table 2). We downloaded version 6 data products derived from the Special Sensor Microwave/Imager instrument [55]. Data processing (monthly averaging and sub-setting) and mapping for all the years (2004–2008) was done in the Sea-WiFS Data Analysis System (SeaDAS) [56] version 5.3, ESRI’s ArcGIS 10 (https://www.esri.com) and the Generic Mapping Tools (GMT) version 6.0.0 [57].

The 4-dimensional variational data (4D-VAR) assimilation approach consists of a fully 3-dimensional space varying parameters and a 1- dimensional time evolving dataset, which determines a model path that best fits observations and factors in time dependent information. Data generated using the 4-dimensional variational ocean data assimilation system [58, 59] can provide vertical temperature and salinity estimates, and mitigate the challenge of having to sample sub-surface waters at the fishing locations. To assess the vertical oceanographic environment at skipjack tuna fishing locations, we used mixed layer depths (MLD) defined on the basis of the density difference (~0.1 σ*θ*) relative to the surface [60] as well as the 4D-VAR derived sub-surface temperature (SST*u*) layers. This approach can provide information on the vertical environment where catches were positive, even though not as highly resolved as data from freely moving tagged fish. We further used histograms to look at the temporal variability in MLD at the gridded fishing locations. Temperatures at depths above 500m, derived from the 4D-VAR data were sampled using the monthly resolved gridded fishing locations. Modelled mean monthly depth-temperature profiles were plotted to assess the vertical temperature profile at the 2004 fishing locations.

### ENFA modeling

**Fig 2:**
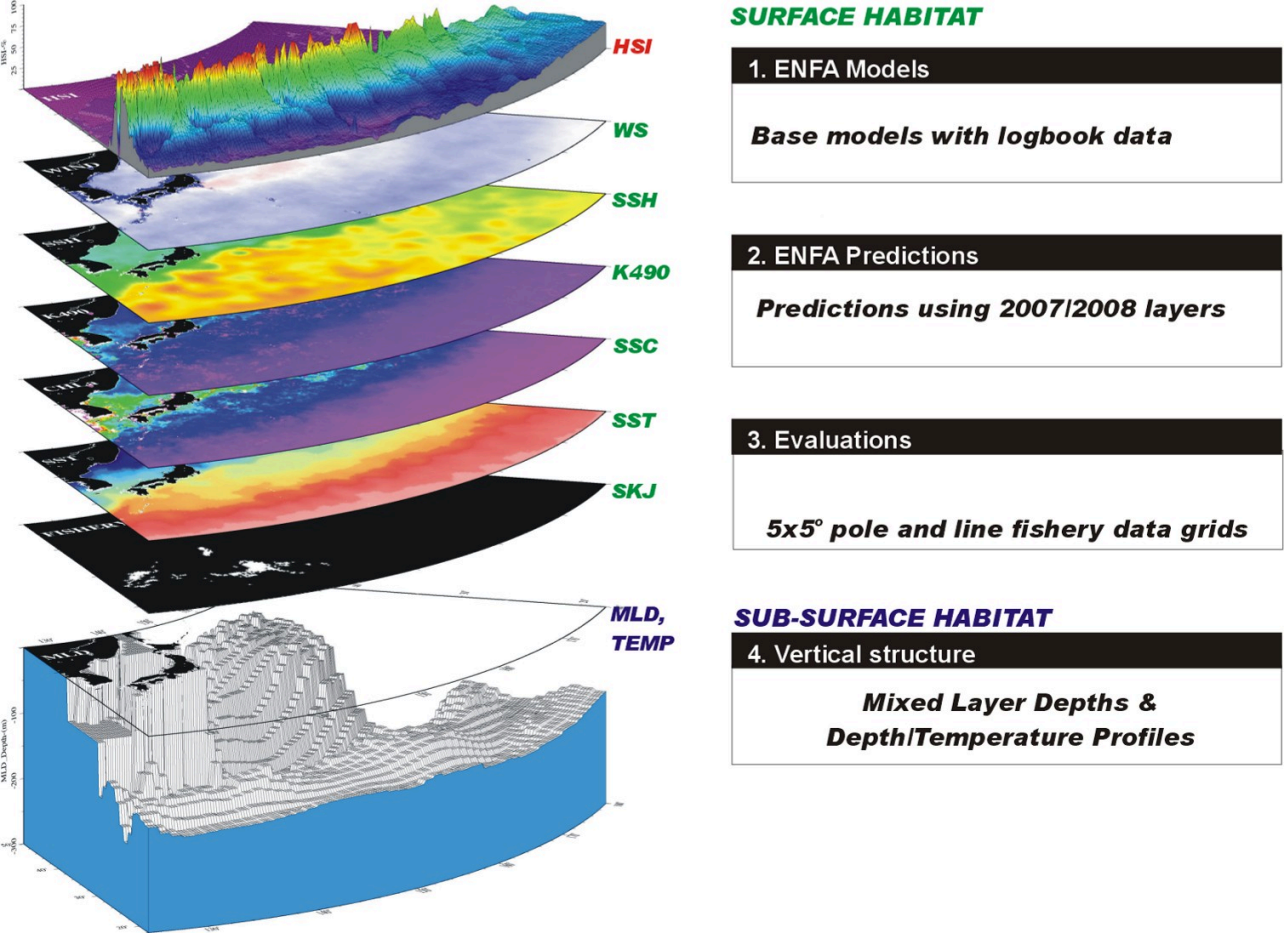
A graphic representation of the workflows and analysis processes.

ENFA modeling of skipjack tuna habitats was conducted in two stages, where base models using gridded fishery data were constructed in the first stage and subsequently used to predict habitats in the second stage. Evaluation of model performance in ENFA was also conducted in the first stage. Our analysis workflows are illustrated in Fig 2. The ecological niche factor analysis (ENFA) is a multivariate technique which uses occurrence records to compute habitat suitability by comparing the species distribution in the environmental space, with that of the whole set of cells potentially available to the species [21]. ENFA modeling requires: (i) the occurrence map (binary) for the focal species in a set of sampled locations and (ii) the independent variables, referred to as eco-geographical variables (EGV), which quantitatively describe the characteristics of each cell [21]. ENFA, uses the marginality and specialization factors, calculated as shown in Eqs 1 and 2. Marginality refers to the ecological distance between the species optimum and the mean habitat within the reference area while specialization refers to the ratio of the ecological variance in mean habitat to that observed for the focal species [21]. Marginality is the first factor extracted in factor analysis for each variable used in the model, while specialization is reflected in subsequent factors.

$$Marginality=|m_{G}‐m_{S}|/1.96\sigma_{G};$$ (1)

$$Specialization=\sigma_{G}/\sigma_{S}$$ (2)

where *m*_*G*_ = global (entire range of cells available to the species in the study area) mean; *m*_*S*_ = species mean; and *σ*_*G*_ = standard deviation of global distribution_;_ *σ*_*S*_ = standard deviation of focal species.

Interpretation of marginality and specialization is based on factor coefficients which give the importance of each variable to the different factors and the range of the environmental values preferred by the species. For marginality, positive coefficients indicate preference for areas above the mean for the variable under consideration while negative coefficients show a preference for areas below the mean [26]. For specialization, the higher the absolute value, the more restricted is the range of the focal species on the corresponding variable. The inverse of specialization is referred to as tolerance. When tolerance values are close to zero, the species tends to live in a narrow range of conditions, while values close to one indicate a species whose habitat preferences within the reference area are broad [21]. The sign on the first factor coefficients is important but only absolute values are considered in the specialization factor coefficients. Factor coefficients are also used to compute global marginality and specialization. The global marginality factor varies between zero and one, with values close to one meaning the species prefers areas with conditions that differ from the average conditions in the reference area. Global specialization can be used for inter-species comparisons, as long as the same area is used as a reference set [21].

For each set of monthly EGVs and the fishery data layer representing presence-only grids for skipjack tuna, we imported the layers into Biomapper 4.0 [61] where maps were verified to ensure that all cells containing valid data were distinguished from those that contained “no-data”. This step was important because we used ocean color data layers which often have missing data due to clouds. Subsequently, factors were computed, a process where ENFA reduces the original set of variables to a subset of uncorrelated factors [21]. The broken-stick rule [62] was used to determine how many of the factors were retained in the habitat suitability calculation. According to the rule, the distribution of the eigenvalue of each factor is compared with the distribution of MacArthur’s broken-stick. The eigenvalues that are larger than expected may be considered ‘significant’ [29, 63]. The retained factors explain most of the information related to the distributions of the original variables and constitute dimensions of the environmental space for calculation of habitat suitability [29]. Whenever ENFA encounters cross-correlated variables, one of them has to be dropped from the model. The geometric mean algorithm was used to compute habitat suitability maps. It generates a smooth set of habitat suitability envelopes by relating each observation cell in such a way that the denser these are in environmental-space, the higher the habitat suitability [61]. A habitat suitability index (HSI) shown as zero indicates the least suitable combination of values for all variables, hence poor habitat. On the contrary, a HSI shown as 100 indicates the most suitable combination of environmental variables, hence the best habitat.

Model performance evaluation was done through cross validation and our Biomapper parameters were set to 10 partitions, a moving window size of 20 bins, with equal bin width, and mean and standard deviation. The performance metrics implemented in Biomapper and used in our work were: the absolute validation index (AVI), the contrast validation index (CVI), the continuous Boyce Index (CBI) and associated predicted-expected (P-E) ratio curves [64]. The AVI is the proportion of presence evaluation points falling above some fixed habitat suitability threshold (e.g. 0.5) and it varies from 0 to 1, while the CVI is the difference between the AVI and the AVI of a model predicting presence everywhere (chance model), and varies from 0 to 0.5 [64]. The higher the AVI and CVI values, the better the model. The CBI is a modified Boyce Index [65] which is computed on the basis of a ‘moving window’ compared to fixed classes [64, 66]. Computation of a CBI starts with a first class covering a defined suitability range whose P-E ratio is plotted against the average suitability value of the class, a process that is repeated by shifting the moving window and plotting the P-E values until the moving window reaches the last possible range [64]. This provides a smooth P-E curve, which is used to generate the CBI. The P-E curves provide three levels of information on model accuracy. First, they show the variance among the cross-validation curves giving information about model robustness all along the HS range. The narrowness of the confidence interval along the curve reflects the model sensitivity to particular calibration points. Second, the shape of the P-E curve is also important, with monotonically ascending slopes indicating a good model (i.e. the P-E value increases proportionally with the habitat suitability). A flat or negative slope indicates an inaccurate model, which predicts poor quality areas where species presences are more frequent. Third, the maximum value reached by the P-E curve reflects how much the model differs from chance expectation, or deviation from randomness, thus indicating the model’s ability to differentiate the species niche characteristics from those of the studied area [64]. The CBI values vary from -1 to 1, with positive values indicating high correlation between increasing P-E and predicted habitat suitability, thus good model calibration. When CBI values are close to zero, model calibration is no better than random. In addition, negative CBI values indicate poor model calibration with inconsistencies between model predictions and locations of validation points [64]. All monthly models generated in ENFA using the 2004 dataset (the base models) were subsequently applied to make predictions using similar monthly averages for corresponding months in 2007 and 2008. Qualitative evaluation of the model predictions in subsequent years was done by overlaying the 5x5 degree fishery polygon data on predicted HSIs.

## Results

### ENFA models

**Fig 3:**
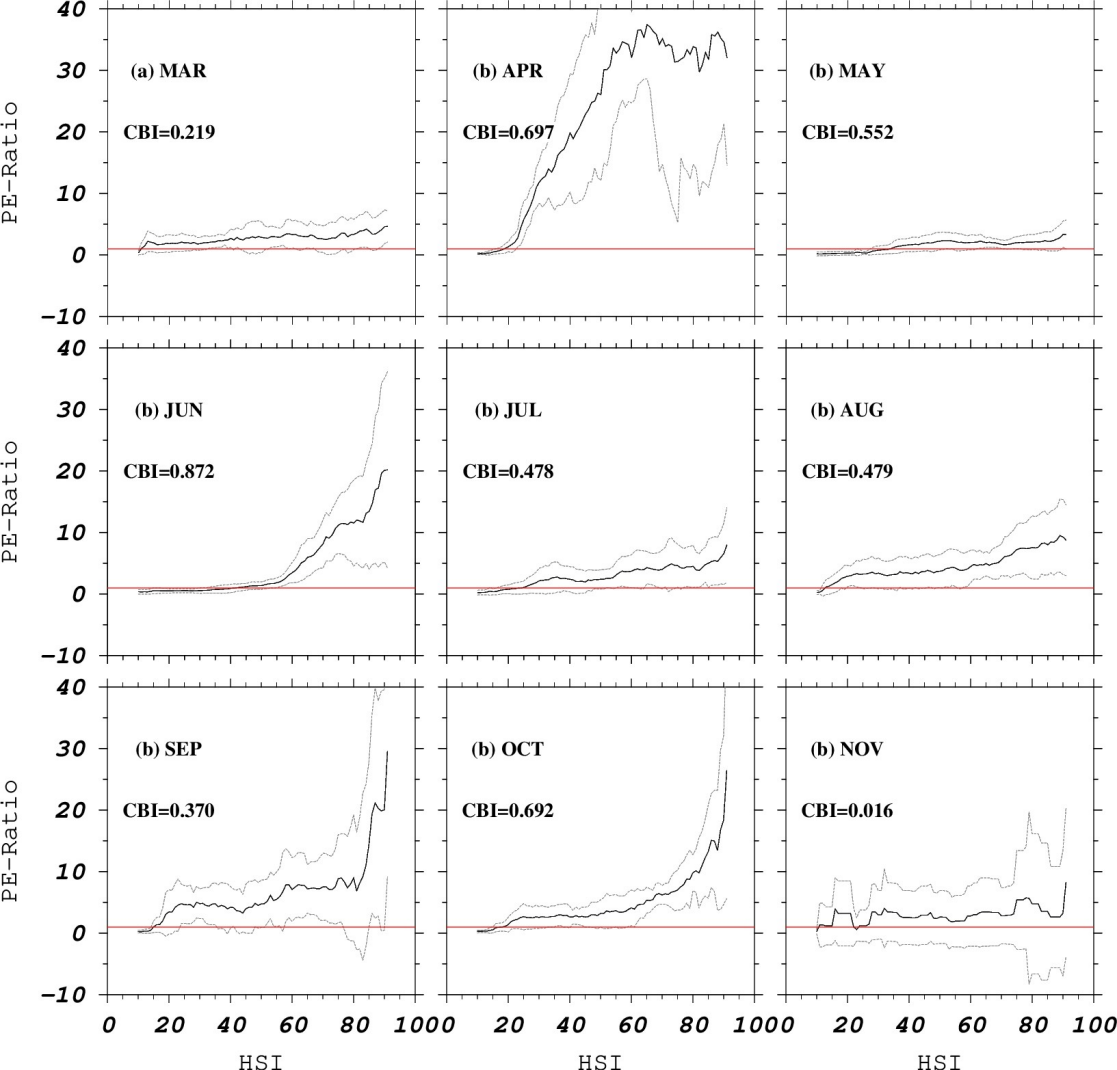
Predicted-expected (P-E) ratios for the base models (March to November 2004), and the respective Continuous Boyce Index (CBI) values for each model.

Global marginality factors obtained from ENFA models were above 0.5 (except for July), while specialization factors were all above 1 (Table 3), pointing to utilization of habitat that was different from the average conditions in the western North Pacific. The global marginality factor is the lowest in July and the highest in September and November while the global specialization is lowest in October and highest in August. The tolerance factors range between 0.122 (August) and 0.452 (October). The marginality factor coefficients (F1) show a strong relationship of skipjack tuna locations with SSH from March to June, when coefficients are positive, and July to November, when coefficients are negative (Table 4). A strong effect of SSH and wind speed in April, 2004 was noted. The contribution of Kd490 is highest in July, October and November. The Kd490 has a low contribution to marginality factor in April, June, August and September. The Kd490 layer was removed from model constructions for March and May due to high cross correlations with SSC, and hence results for this variable in those models are not shown. The contribution of variables to specialization factors (F2-5) varies considerably. However, the importance of chlorophyll-a and/or SST can be seen in many of the factors, especially the first and second specialization factors (F2 and F3). The model performance metrics (AVI, CVI and CBI) indicate relatively good models, except for the March and November models, when the CBI is considered. All the AVI and CVI values are positive (Table 3). All the P-E curves portray positive and monotonic ascending slopes, except for the November curve which is almost flat for all the suitability scores, and also shows wide confidence intervals (Fig 3).

**Table 3:** Global Marginality (M), Specialization (S) and Tolerance (S) factors derived from skipjack tuna ENFA models.

Month	M	S	T	*AVI*	*AVI-SD*	*CVI*	*CVI-SD*	*CBI*
MAR	0.691	7.507	0.133	0.504	0.250	0.367	0.246	0.219
APR	0.564	5.992	0.167	0.482	0.098	0.462	0.098	0.697
MAY	0.602	4.897	0.204	0.570	0.245	0.341	0.236	0.552
JUN	0.502	6.624	0.151	0.527	0.285	0.448	0.263	0.872
JUL	0.455	6.194	0.161	0.506	0.351	0.405	0.344	0.478
AUG	0.527	8.166	0.122	0.456	0.244	0.382	0.239	0.479
SEP	0.729	5.828	0.172	0.532	0.277	0.489	0.274	0.370
OCT	0.662	2.211	0.452	0.532	0.242	0.464	0.235	0.692
NOV	0.778	5.195	0.195	0.550	0.445	0.462	0.435	0.016

The absolute validation index (AVI), contrast validation index (CVI) values and their respective standard deviations, and the continuous Boyce index (CBI) value for each monthly model.

**Table 4:** Contribution of the variables to the factors (F) generated by ENFA and used to build the monthly habitat suitability maps.

**Month**	**Variable**	**F1 (81%)**	**F2 (13%)**	**F3 (5%)**	**F4 (2%)**	**F5 (%)**
MAR	SSH	0.77	0.31	0.62	-0.58	-
	SST	0.63	-0.62	-0.71	0.73	-
	WIND	0.03	0.03	-0.31	-0.37	-
	CHLA	-0.20	-0.72	0.15	-0.07	-
	K490	-	-	-	-	-
		**F1 (61%)**	**F2 (31%)**	**F3 (6%)**	**F4 (1%)**	**F5 (0%)**
APR	SSH	0.67	0.02	-0.12	-0.10	0.59
	WIND	0.61	-0.04	-0.03	-0.32	-0.17
	SST	0.30	0.52	0.16	0.27	-0.73
	K490	0.29	-0.57	0.28	0.70	-0.27
	CHLA	0.03	0.64	-0.94	-0.57	0.14
		**F1 (43%)**	**F2 (46%)**	**F3 (9%)**	**F4 (4%)**	**F5 (%)**
MAY	SSH	0.91	-0.12	0.34	0.20	-
	SST	0.33	-0.39	-0.93	-0.26	-
	WIND	0.01	0.01	-0.13	0.86	-
	CHLA	-0.26	-0.91	0.00	0.38	-
	K490	-	-	-	-	-
		**F1 (36%)**	**F2 (49%)**	**F3 (14%)**	**F4 (1%)**	**F5 (0%)**
JUN	SSH	0.83	-0.07	0.15	0.28	0.46
	K490	0.39	-0.08	0.37	-0.17	-0.48
	SST	0.27	-0.51	-0.91	-0.74	-0.62
	WIND	0.11	-0.02	-0.03	0.58	-0.40
	CHLA	-0.28	-0.85	0.06	0.08	-0.08
		**F1 (73%)**	**F2 (20%)**	**F3 (6%)**	**F4 (0%)**	**F5 (0%)**
JUL	K490	0.82	0.24	0.22	0.55	-0.42
	SST	0.07	-0.04	-0.78	0.05	0.27
	CHLA	-0.16	0.97	-0.44	0.23	0.11
	WIND	-0.17	-0.02	0.03	0.47	0.36
	SSH	-0.51	0.08	0.39	0.65	-0.78
		**F1 (33%)**	**F2 (54%)**	**F3 (9%)**	**F4 (2%)**	**F5 (1%)**
AUG	K490	0.14	-0.54	-0.51	0.83	-0.79
	WIND	0.06	-0.02	-0.11	0.35	0.16
	CHLA	-0.01	0.83	0.26	-0.38	0.53
	SST	-0.22	0.12	-0.80	0.17	-0.25
	SSH	-0.96	-0.12	0.10	0.11	-0.05
		**F1 (17%)**	**F2 (58%)**	**F3 (17%)**	**F4 (7%)**	**F5 (1%)**
SEP	K490	0.33	-0.63	-0.32	0.14	0.85
	CHLA	0.17	0.70	0.45	-0.44	-0.40
	WIND	-0.02	0.02	-0.35	-0.46	0.06
	SST	-0.44	-0.34	0.66	-0.68	0.32
	SSH	-0.82	0.07	-0.38	0.34	0.09
		**F1 (14%)**	**F2 (44%)**	**F3 (30%)**	**F4 (8%)**	**F5 (4%)**
OCT	K490	0.49	-0.57	-0.53	0.79	-0.72
	CHLA	0.23	0.76	0.55	-0.46	0.06
	WIND	-0.09	-0.04	-0.05	-0.17	-0.45
	SST	-0.35	-0.30	0.51	0.11	-0.51
	SSH	-0.76	0.01	-0.40	0.34	-0.16
		**F1 (35%)**	**F2 (48%)**	**F3 (13%)**	**F4 (2%)**	**F5 (1%)**
NOV	K490	0.56	-0.55	-0.20	-0.13	0.75
	CHLA	0.33	0.82	-0.17	0.19	-0.37
	WIND	0.05	0.06	-0.20	0.97	0.13
	SSH	-0.65	0.05	0.27	0.00	0.52
	SST	-0.39	-0.17	-0.90	0.10	-0.08

The marginality factor coefficients are shown on the first factor (F1), against the respective variable. Factors 2–5 represent specialization factors. Specialization values for each factor are shown in parentheses. The coefficients are sorted by decreasing order of the first factor (marginality).

### Habitat suitability and fishing activities

**Fig 4:**
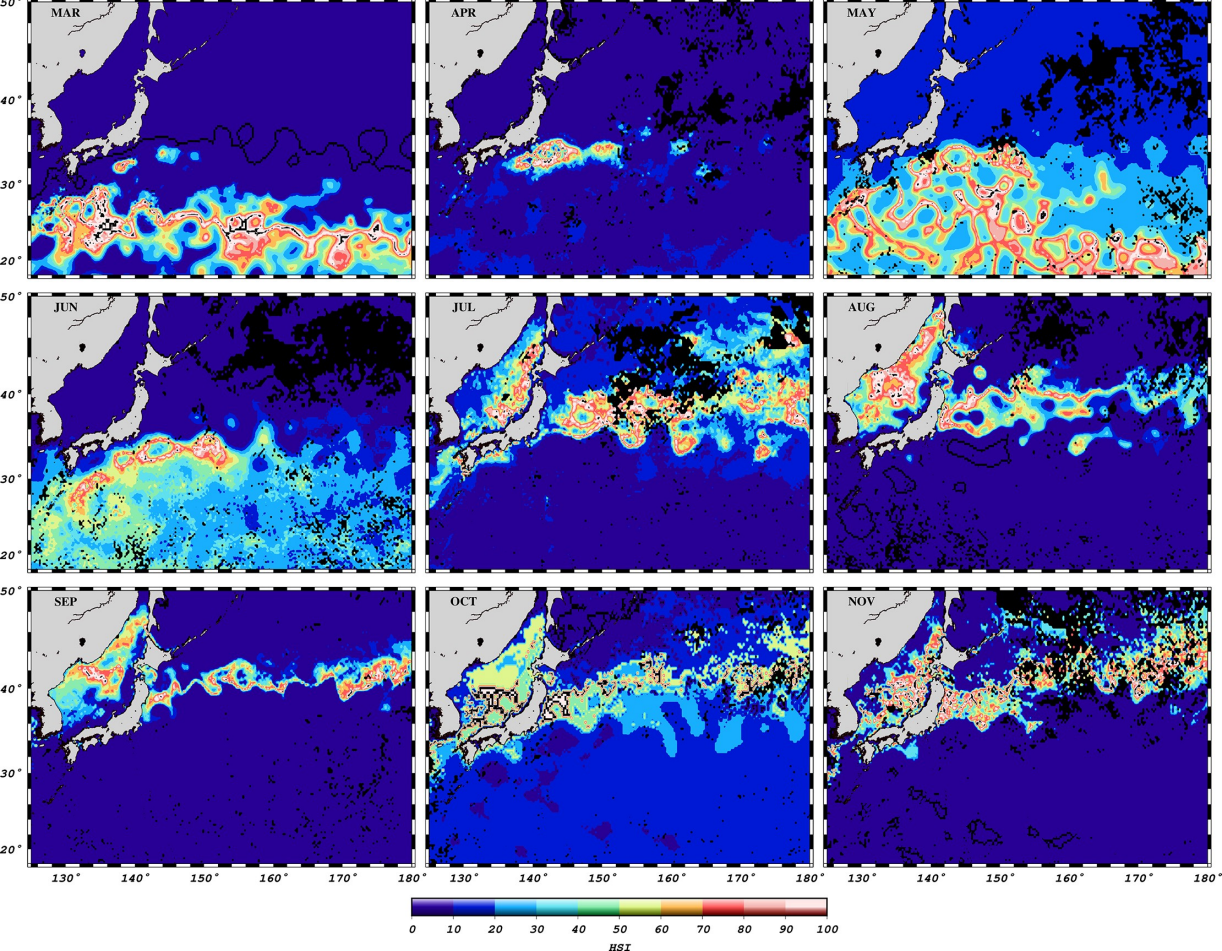
Habitat Suitability Indices (HSI) for the base models, from March to November (2004).

**Fig 5:**
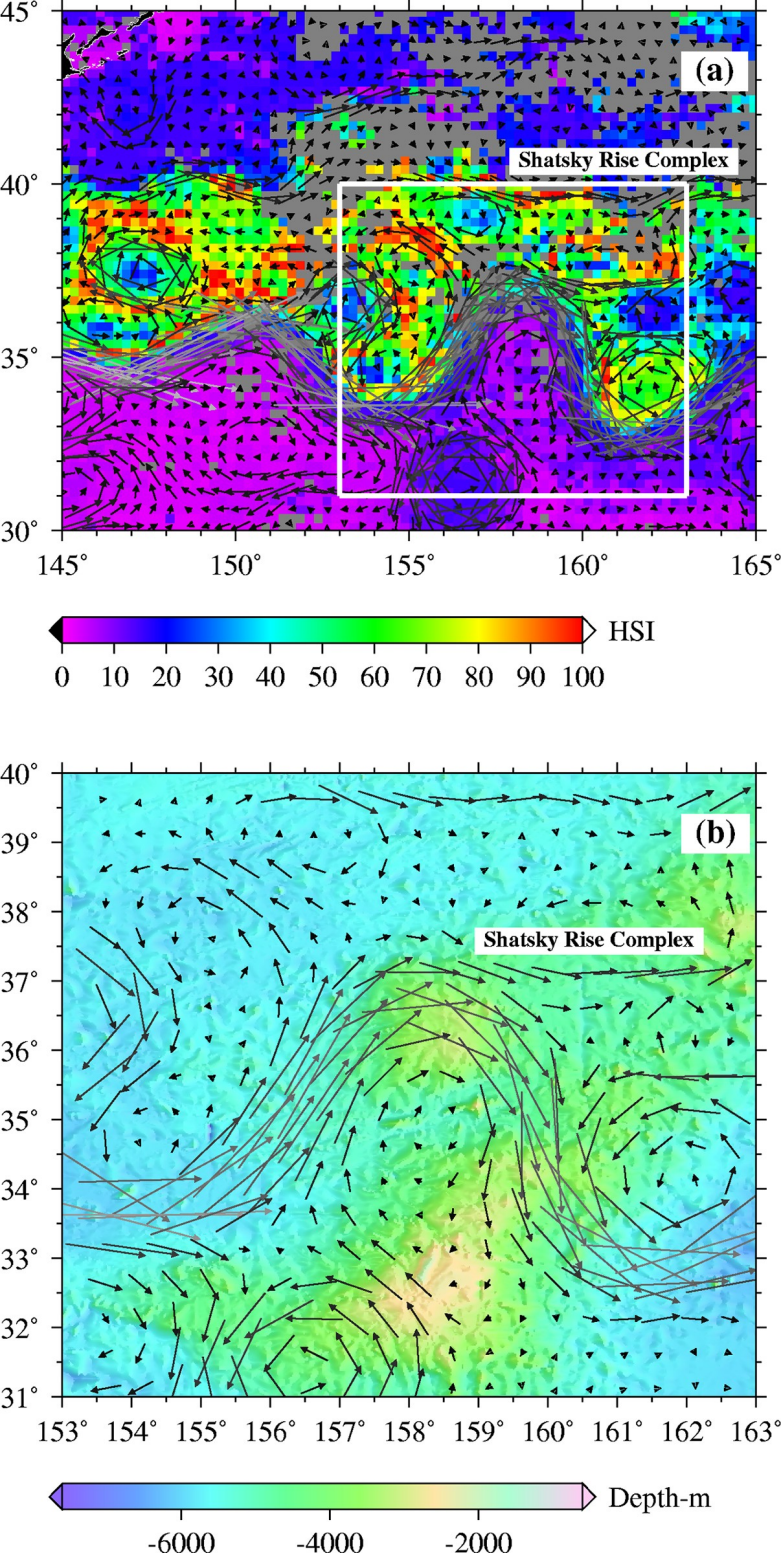
The Shatsky Rise area, (a) the surface flow of the Kuroshio Extension in July 2004, and the high habitat suitability indices (HSIs) north of the Shatsky Rise, and (b) the impact of the bathymetry around the Shatsky Rise area, on the surface currents in July 2004.

**Fig 6:**
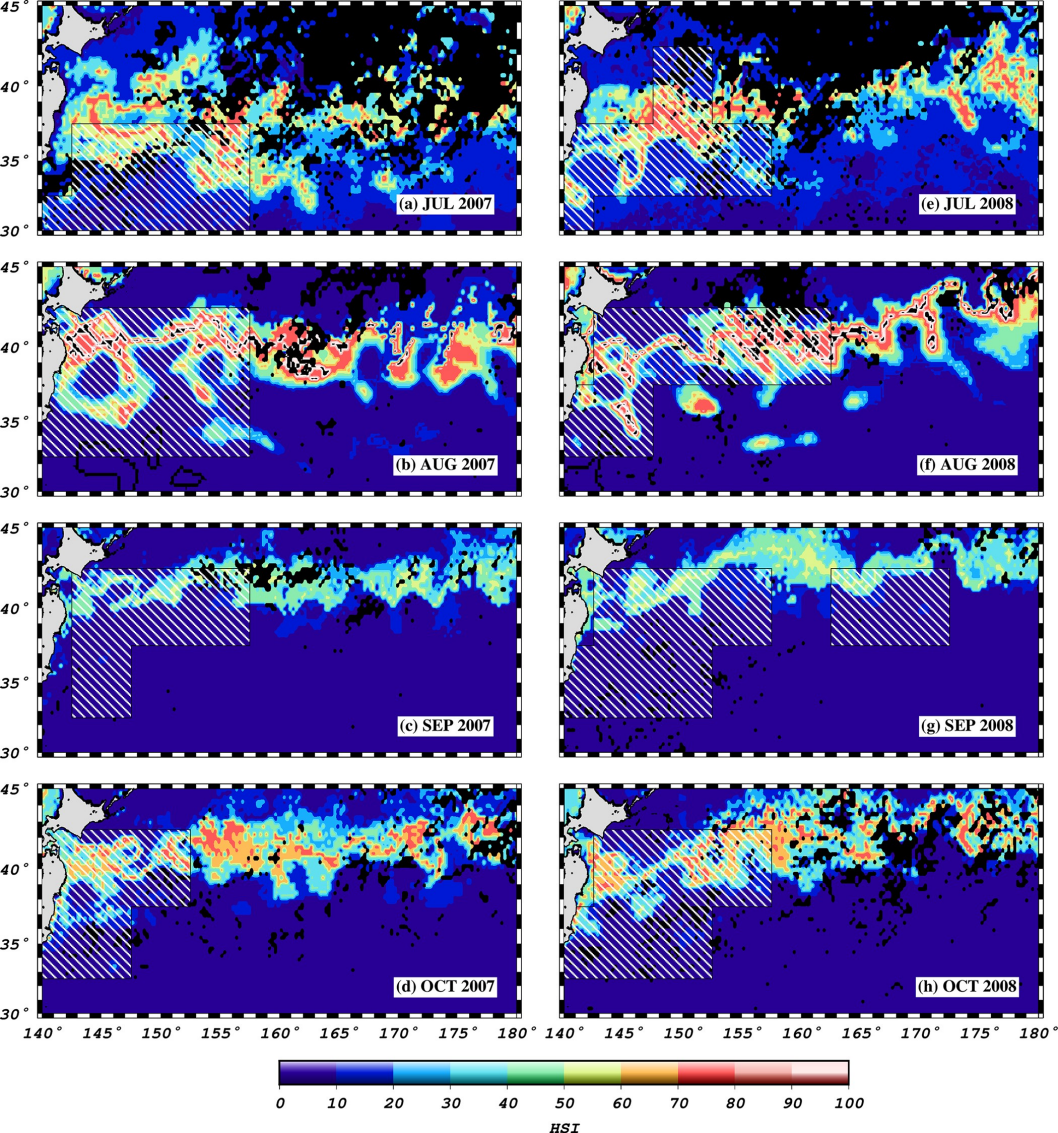
Habitat suitability indices predicted for July-October, 2007 and 2008, (a-h) overlaid with polygons (white shaded polygons) derived from 5x5 degree grids indicating areas fished in the respective months.

The base model of habitat suitability revealed high scores from 25^o^N in March to approximately 41^o^N in September/October (Fig 4). Habitat hotspots are spread expansively in March, May and June. From July to November, formation of habitat hotspots for skipjack tuna is characterized by rather thin “strips” of high suitability scores. The effect of meandering and eddies pinched off the Kuroshio Extension, on habitat formation can be observed in Fig 5A. High suitability scores occurred along an eddy north of the Kuroshio Extension, and along currents spinning off the Kuroshio Extension. The effect of the underlying Shatsky Rise Complex on the oceanographic circulation is illustrated with monthly averaged geostrophic velocity vectors (Fig 5B). The 5x5 degree pole and line fished area polygons show congruence with predicted habitats (Fig 6) in some areas and not others (e.g. September 2007 and 2008).

### Sub-surface environment variability

**Fig 7:**
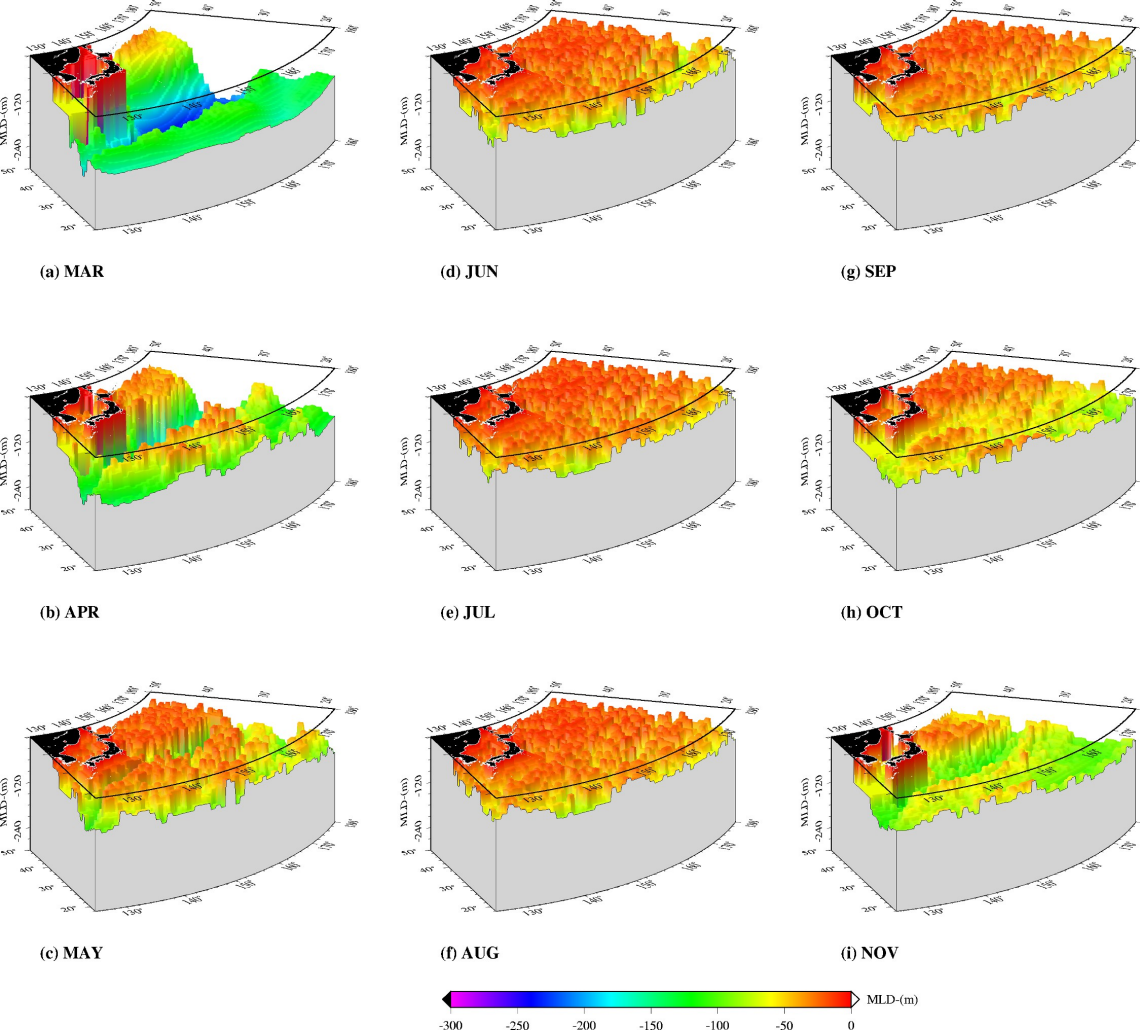
Monthly changes in mixed layer depths (MLD) in the western North Pacific, March to November, 2004.

**Fig 8:**
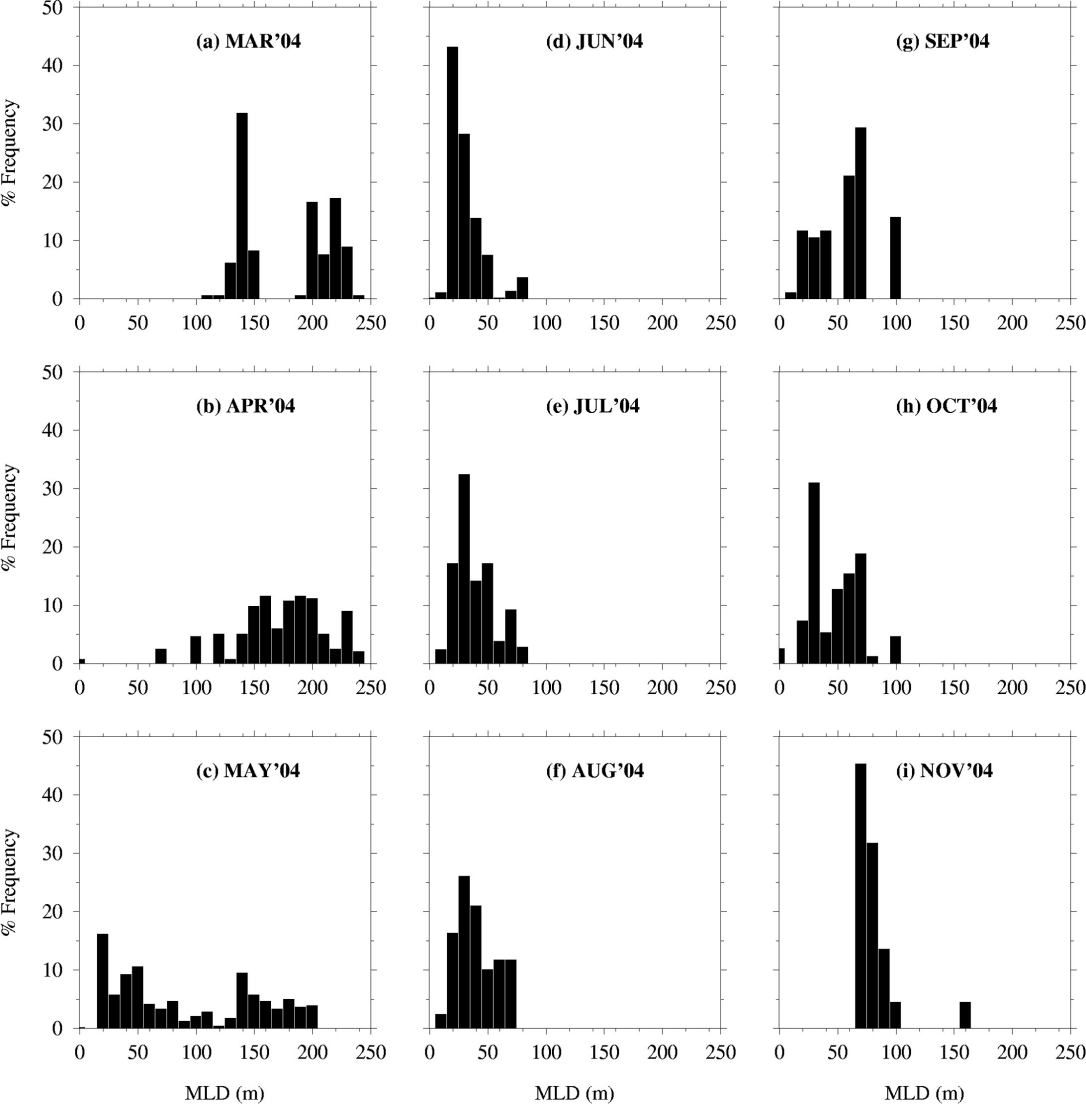
Histograms of mixed layer depths (MLD) sampled at fishing locations, March to November, 2004.

**Fig 9:**
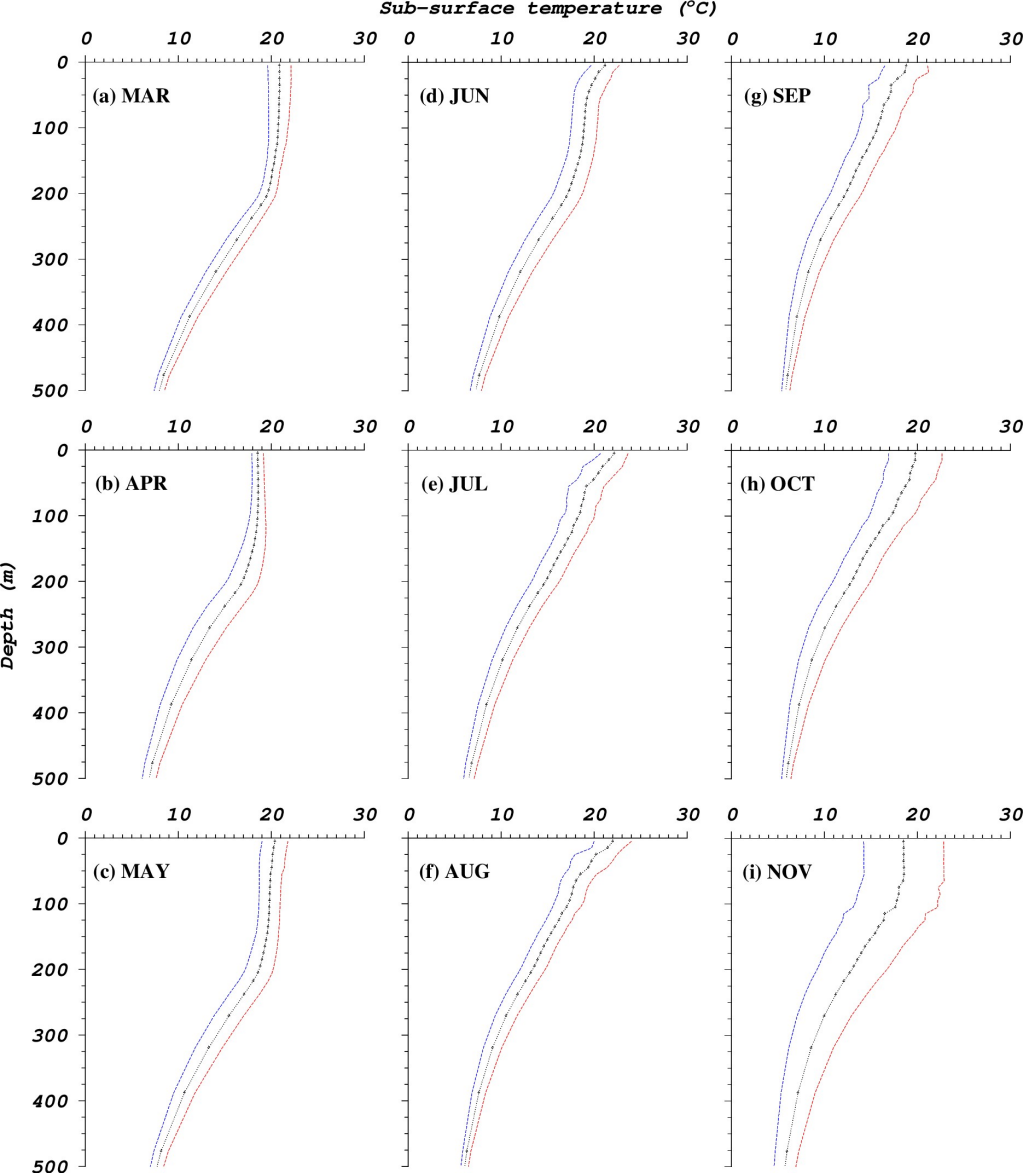
Depth-temperature profile illustrated as mean monthly temperature (derived from 4D-VAR generated data) at fishing locations. Dashed lines illustrate ±1 standard deviation from the mean.

**Fig 10:**
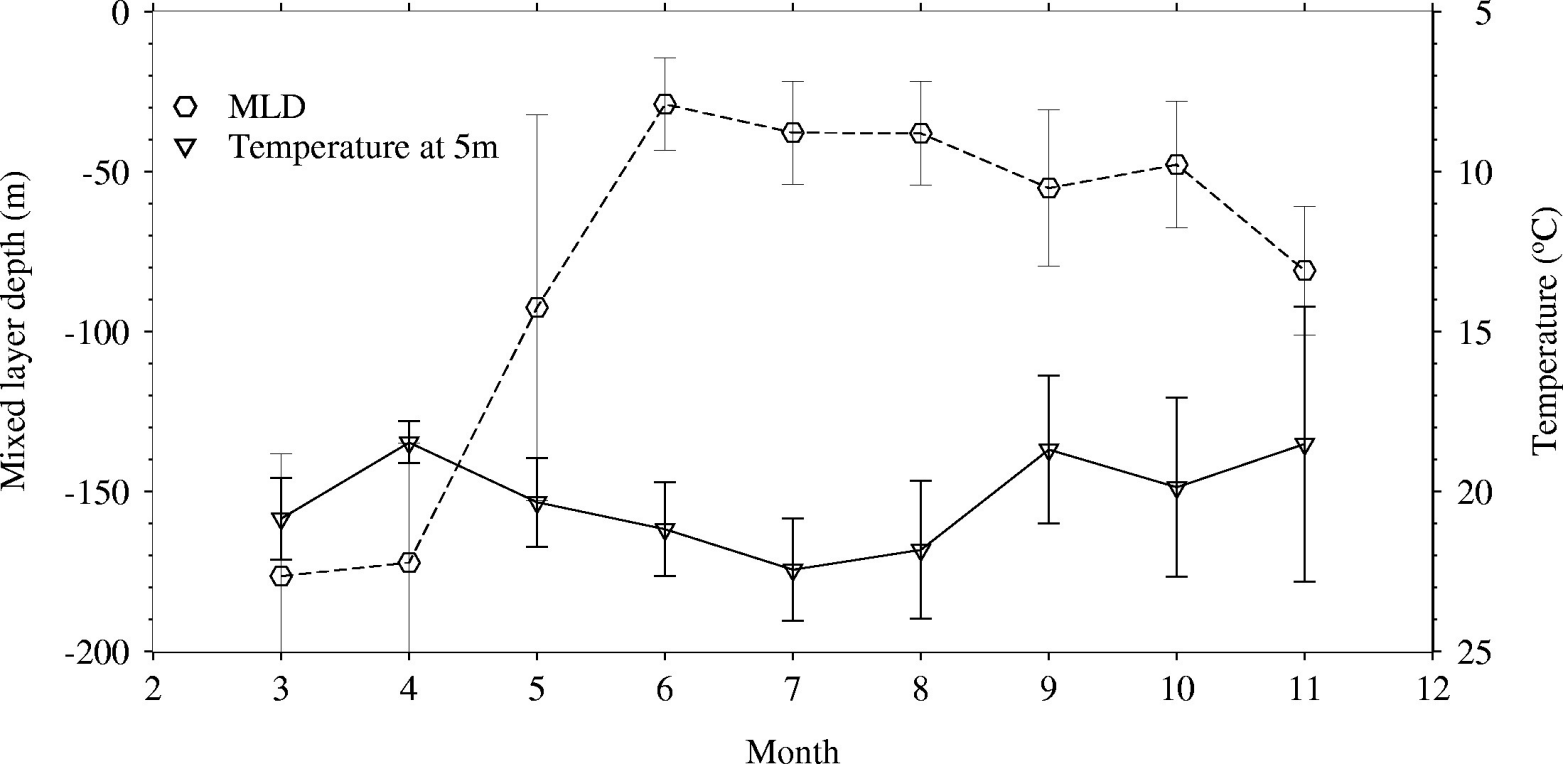
Mean monthly mixed layer depths (MLD) and temperature at 5m at fishing locations, March to November, 2004.

Mixed layer depths in the western North Pacific in 2004 were deepest in winter and early spring (March-April) and shallowest in summer (June-August) (Fig 7). Some of the fishing locations in areas with deepest MLDs have values above 200m (March-May) while the shallowest values lie between 10m and 100m (e.g. June-August) (Fig 8). From June to October, fishing occurred in areas where MLDs were below 100m. Depth-temperature profiles derived from temperatures averaged at fishing locations corresponding to the various 4D-VAR model data layers show changes from a homogenous temperature layer (March-May) to one that stratifies from June to October 2004 (Fig 9). November shows a re-establishment of the homogenous water column. Between March and July, mean temperatures are above 15°C within the 200m surface layer. However, from August to November, some depths show mean temperatures below 15°C, within the 200m surface layer. Fig 10 emphasizes the seasonal variability in mean monthly MLD relative to mean monthly temperatures at 5 meters (at fishing locations), where temperatures in summer are associated with shallow MLDs.

## Discussion

### ENFA models

To understand skipjack tuna’s habitat hotspots in the western North Pacific, we constructed models using fishery presence-only data and five satellite remotely sensed variables, in Biomapper 4.0, following the ENFA approach. ENFA’s model performance metrics (AVI, CVI, CBI and P-E curves) indicated that the April to October models performed well, and thus had relatively good predictive power. The low CBI values for March and November models indicate low-quality models which could be attributed to inadequate occurrence samples in these months given that these two periods represent the initial and last time spans of the fishing season for skipjack tuna in the study area respectively. It has been shown that small presence samples can affect model calibration and evaluation [64]. In addition, the March and November models have high average marginality values, implying that skipjack tuna in these months occur in zones that differ significantly from the rest of the study area. This observation can also be explained by skipjack tuna’s migratory behavior in relation to sea surface temperature. From early spring, skipjack tuna are known to migrate north and track warm waters above 18°C, while in late autumn (November), the northern migration is hampered by surface cooling of the Oyashio area [67], and thus the species is quite selective in utilization of space, in relation to spatial temperature distribution.

The observed seasonal northward displacement of skipjack tuna habitat hotspots in the entire study area (Fig 4) is characteristic of the latitudinal migration of skipjack tuna [68, 69]. The migration is closely associated with seasonal warming and sea surface temperature and chlorophyll-a concentration gradients, [8, 19, 70]. Thermal and ocean color gradients are important indicators of skipjack tuna fishing grounds in the western North Pacific, and often point to areas of elevated productivity which attract tuna, as they forage on lower trophic level organisms [7]. The hotspot formation for April (2004) was located around the Kuroshio front, an outcome that portrays the effect of aggregation of fishing locations around a major SST front (Fig 4). This implies that the ENFA computed variable means were very similar to values around the frontal zone (and quite different from the rest of the study area), thus limiting the model’s capability to “pick” areas outside the domain for which fishing data were available. The formation of habitat hotspots in June and July (base models), between 30-35^o^N and 150-160^o^E, the area on or around the Shatsky Rise is another region where ENFA indicates high suitability scores for skipjack tuna occurrence. The main Shatsky Rise and the larger Shatsky Rise complex (Fig 5) affect the circulation of the Kuroshio Extension [71] and the mechanisms through which this feature enhances the magnitude and transfer of primary production to top predators such as tuna is well documented [3, 72]. The ENFA marginality and specialization factors support this view, by indicating that hotspot conditions differ markedly from the average conditions in the larger study area.

### Habitat suitability and fishing activities

The ENFA models indicated the importance (higher values of first factor) of Kd490 when fishing locations were situated in the Kuroshio-Oyashio Transition Zone (July, October and November) compared to the Kuroshio area (March to June). In the Kuroshio area where waters are warm and oligotrophic, Kd490 had little influence on habitat models, compared to the 3 months (July, October and November), when fishing occurred in the mixed zone, where waters are more turbid and productive. Previous work has indicated that turbidity influences tuna distribution [53], and skipjack tuna are attracted to productive waters because they forage on small organisms (small fishes, squids) whose forage requirements are met by dense plankton blooms [12, 17]. However, they remain in waters whose temperature is physiologically tolerable, and visibility is good.

Our results showed positive first factor coefficients for SSH when the fishing locations are within Kuroshio waters (warmer and lower chlorophyll-a), and negative first factor coefficients when in the mixed zone (cooler and higher chlorophyll-a). This can be attributed to the differences in SSH values between these two regions, which are oceanographically very distinct. The sea surface height data are useful in identifying ocean currents and cold and warm core eddies. For the western North Pacific, the Kuroshio and Oyashio currents, and eddies pinched off from these currents have distinct SSH signatures that are useful as indicator variables for hotspot modelling [3]. The edges of large warm core eddies, easily identifiable with SSH data, are known to provide good fishing grounds for skipjack tuna [17].

The ENFA model results indicate that the first factor coefficients for the wind variable were low for all months except April, 2004. The surface wind speed data were used in this work mainly for two reasons. First, surface winds driven by typhoons and hurricanes cause mixing of the upper mixed layer, which can induce upwelling and cause elevated productivity [73]. Such elevated productivity can lead to further downstream aggregation of tuna. Second, during extremely windy conditions, tuna fishing can be hindered by bad weather. Consequently, fishery data can show temporal gaps in certain areas simply because vessels were not able to fish under bad weather conditions [74]. While both reasons are valid, surface wind induced productivity and bad weather considerations may not have direct effects on tuna habitats, compared to the other variables. In addition, the frequency of these two events during a fishing season maybe low, and therefore had little influence in selection of fishing locations by fishermen, which consequently expresses as minimal contribution in ENFA models and HSI computation.

The spatial congruence between predicted hotspots (July-October; 2007 and 2008) and areas fished (as indicated by the 5x5 degree data) point that ENFA models correctly predicted some of the areas that were indeed fished, or where skipjack tuna were present. This shows that the ENFA models and the datasets used can be applied successfully to predict potential habitat hotspots or fishing zones for skipjack tuna. The discrepancies in predicted areas and mapped polygons might be explained by possible inability by the ENFA models to predict all potential areas, and the coarseness of the 5x5 degree data from which the mapped polygons were derived. Given that the ENFA models were constructed using a much finer resolution occurrence dataset, comparing the model output to a polygon derived from a coarser resolution dataset was bound to show some discrepancies.

### Sub-surface environment variability

Sub-surface temperature and mixed layer depth data generated by an ocean circulation model, were used to determine sub-surface conditions around the fishing locations. Two approaches can be used to remotely measure ocean sub-surface habitats utilized by tuna, ocean circulation model data and data from tagging experiments. Tags are often expensive and deployment can be costly and labor intensive, thus limiting the numbers that can be deployed at a given time. In the absence of information from tagging experiments, data from general circulation models are useful for indicating the sub-surface conditions under which fish were caught, and also for improving synoptic coverage of vertical habitat utilization [75]. This approach improves our knowledge of the horizontal and the vertical habitat. The results of such work are important in improving fishery forecasting models in the western North Pacific. Skipjack tuna are pelagic fishes and the pole and line fishery targets fish within the upper mixed layer of the water column where they confine in waters high in dissolved oxygen [67, 76]. The upper mixed layer is affected by surface warming, which influences the depth, temperature, and primary production in the mixed layer [32]. Understanding how a warm-water species like skipjack tuna responds to seasonal temperature variations in the upper mixed layer can provide insights into their biological responses in a warmer ocean [77, 78]. First, from the MLD data (Fig 7), the seasonal shoaling of the mixed layer from spring to summer and deepening in winter is as expected for the western North Pacific [79–81]. Second, the seasonal shoaling of MLDs is associated with the seasonal warming, which raises the sea surface temperatures from spring to summer, when the fish migrate north. The shoaling of the thermocline compresses the depth (Fig 8) at which tuna have access to abundant food, resulting in increased vulnerability of the fish to surface fisheries [82]. The depth of the mixed layer (Fig 8) affects the temperature of the mixed layer [83, 84], the amount of solar insolation available to phytoplankton cells [54], and the quantities of nutrients available for photosynthesis [85], which in turn affects primary production, and by extension secondary production. Model predictions indicate that mixed layer depths are likely to be shallower in the 21^st^ century, as the western North Pacific Ocean warms up [80, 81]. As a result of surface warming, the western North Pacific is expected to stratify further (with the MLD becoming shallower) in January and February which would cause the spring bloom to occur earlier [81, 86]. Similar findings show spring blooms [80] and forage biomass [87] will shift northward due to climate change. This is expected to prolong the growing season at high latitudes, driven primarily by increased stratification leading to better photosynthetic efficiency in spring and summer [80]. These findings correspond with projections from a number of models which show a consistent shoaling of the mixed layer in the Kuroshio Extension [88] and expansion of the subtropical biome could lead to an increase in primary production and fish catch [78, 89].

The monthly mean temperature-depth profiles (Fig 9), though not as detailed as those acquired through archival tags which provide diurnal variability of ambient and peritoneal temperatures of tagged fish [32, 90], provide important information on the vertical habitat underlying locations where skipjack tuna were caught. In a study by [90], skipjack tuna in the Kuroshio area were observed to dive to varying depths during the day but in most instances retained the body cavity temperature at around 17°C. In some instances, skipjack tuna swam in waters where temperature dropped to 12°C during deep dives. Our findings on the temperature-depth profiles are consistent with the previous work [32, 90]. Recently, [67] also found that throughout skipjack tuna’s northward migration in the western North Pacific, their vertical distribution became shallower in higher latitudes, which is thought to be a strategy to avoid exposure to the cold water less than 18°C. This information can help managers to assess how these fishes are likely to respond biologically to warming of the upper ocean. In the western North Pacific, skipjack tuna have been caught as far as 44^o^N [15] in summer and autumn seasons when warm waters provide conducive habitat in higher latitudes. The zonal extent of the northward migration could largely depend on how much warming can alter the surface or mixed layer temperatures during the autumn-winter period, as well as the characteristics of the Oyashio Current whose cold waters and winter mixing inhibit northward migration of skipjack tuna [70].

Our work was subject to three limitations which are worth highlighting. First, we used a single-year fishery dataset which limited our ability to make inter-annual comparisons of hotspots variability within the study area. Failure to use a multi-year dataset can only be explained by our inability to access more data due to logistical and administrative processes at the time the work was conducted, and not unavailability of the data. Future work using a multi-year dataset would therefore expand the scope of our work by extensively analyzing the inter-annual variability of skipjack tuna hotspots, and the effects of ocean and climate variability on hotspot formation. Second, we used chlorophyll-a and Kd490 satellite derived datasets with missing values due to clouds. The missing data values hampered the computation of HSI scores in affected pixels, hence the spatial coverage of HSI in months where cloud coverage was extensive does not present a full picture of hotspot dynamics. Third, our models were computed at 0.25-degree spatial resolution, on a monthly time scale, which were also the spatial and temporal resolutions of our input datasets. We suggest that using higher resolution datasets for smaller zones within our study area could refine hotspot analysis, and certainly improve the utility of the outputs at shorter time-scales.

In summary, based on our findings, we make the following conclusions:

- ENFA models generated and predicted skipjack tuna habitat hotspots, which were consistent with the known distribution ecology and seasonal migration pattern of the species. The good performance of ENFA models was demonstrated by the 4 metrics used to evaluate model quality in ENFA. Consequently, our attempts to predict potential hotspots in subsequent years were successful, and the qualitative comparisons between predicted and fished areas showed correspondence when compared with polygons indicating areas fished for the same period. In addition, we conclude that the models were robust at explaining the ecological importance of the variables used in formation of skipjack tuna habitat hotspots.
- The combined application of diverse datasets and tools (e.g. fishery and satellite datasets, ocean circulation model datasets and ecological niche models) in fisheries oceanography can improve our understanding of pelagic hotspots, thus facilitating better tools for fishing ground prediction and management.

## Supporting information

S1 Table Global correlation coefficients among the 5 variables used. (DOCX)
