# Supplementary material for: Identification of skipjack tuna (Katsuwonus pelamis) pelagic hotspots applying a satellite remote sensing-driven analysis of ecological niche factors: A short-term run
Source: PLoS One. 2020 Aug 20;15(8):e0237742. doi: 10.1371/journal.pone.0237742 (PMC7440647; doi:10.1371/journal.pone.0237742)
Supplement: S1 Table — (DOCX) [file pone.0237742.s001.docx]

**S1 Table. Global correlation coefficients among the 5 variables used.**

|  |  | SST | SSH | SSC | WS | KD490 |
| --- | --- | --- | --- | --- | --- | --- |
| MAR | SST | 1.000 | 0.905 | -0.096 | 0.389 | 0.000 |
|  | SSH | 0.905 | 1.000 | -0.082 | 0.370 | 0.024 |
|  | SSC | -0.096 | -0.082 | 1.000 | 0.009 | 0.990 |
|  | WS | 0.389 | 0.370 | 0.009 | 1.000 | 0.202 |
|  | KD490 | 0.000 | 0.024 | 0.990 | 0.202 | 1.000 |
| APR | SST | 1.000 | 0.918 | -0.365 | -0.577 | -0.279 |
|  | SSH | 0.918 | 1.000 | -0.396 | -0.603 | -0.293 |
|  | SSC | -0.365 | -0.396 | 1.000 | 0.881 | -0.228 |
|  | WS | -0.577 | -0.603 | 0.881 | 1.000 | -0.172 |
|  | KD490 | -0.279 | -0.293 | -0.228 | -0.172 | 1.000 |
| MAY | SST | 1.000 | 0.916 | -0.501 | 0.153 | 0.106 |
|  | SSH | 0.916 | 1.000 | -0.494 | 0.131 | 0.182 |
|  | SSC | -0.501 | -0.494 | 1.000 | -0.382 | 0.995 |
|  | WS | 0.153 | 0.131 | -0.382 | 1.000 | 0.290 |
|  | KD490 | 0.106 | 0.182 | 0.995 | 0.290 | 1.000 |
| JUN | SST | 1.000 | 0.877 | -0.571 | -0.757 | 0.571 |
|  | SSH | 0.877 | 1.000 | -0.481 | -0.768 | 0.583 |
|  | SSC | -0.571 | -0.481 | 1.000 | 0.409 | -0.434 |
|  | WS | -0.757 | -0.768 | 0.409 | 1.000 | -0.657 |
|  | KD490 | 0.571 | 0.583 | -0.434 | -0.657 | 1.000 |
| JUL | SST | 1.000 | 0.873 | -0.588 | -0.782 | 0.080 |
|  | SSH | 0.873 | 1.000 | -0.510 | -0.815 | 0.170 |
|  | SSC | -0.588 | -0.510 | 1.000 | 0.515 | -0.323 |
|  | WS | -0.782 | -0.815 | 0.515 | 1.000 | -0.177 |
|  | KD490 | 0.080 | 0.170 | -0.323 | -0.177 | 1.000 |
| AUG | SST | 1.000 | 0.854 | -0.523 | -0.748 | 0.138 |
|  | SSH | 0.854 | 1.000 | -0.480 | -0.701 | 0.216 |
|  | SSC | -0.523 | -0.480 | 1.000 | 0.906 | -0.308 |
|  | WS | -0.748 | -0.701 | 0.906 | 1.000 | -0.311 |
|  | KD490 | 0.138 | 0.216 | -0.308 | -0.311 | 1.000 |
| SEP | SST | 1.000 | 0.885 | -0.597 | -0.808 | 0.060 |
|  | SSH | 0.885 | 1.000 | -0.536 | -0.746 | 0.115 |
|  | SSC | -0.597 | -0.536 | 1.000 | 0.908 | -0.307 |
|  | WS | -0.808 | -0.746 | 0.908 | 1.000 | -0.280 |
|  | KD490 | 0.060 | 0.115 | -0.307 | -0.280 | 1.000 |
| OCT | SST | 1.000 | 0.896 | -0.592 | -0.800 | -0.049 |
|  | SSH | 0.896 | 1.000 | -0.524 | -0.736 | 0.040 |
|  | SSC | -0.592 | -0.524 | 1.000 | 0.908 | -0.277 |
|  | WS | -0.800 | -0.736 | 0.908 | 1.000 | -0.215 |
|  | KD490 | -0.049 | 0.040 | -0.277 | -0.215 | 1.000 |
| NOV | SST | 1.000 | 0.890 | -0.520 | -0.733 | -0.286 |
|  | SSH | 0.890 | 1.000 | -0.491 | -0.707 | -0.274 |
|  | SSC | -0.520 | -0.491 | 1.000 | 0.893 | -0.225 |
|  | WS | -0.733 | -0.707 | 0.893 | 1.000 | -0.124 |
|  | KD490 | -0.286 | -0.274 | -0.225 | -0.124 | 1.000 |
